# Effects of Functional Foods and Dietary Bioactives on Human Health

**DOI:** 10.3390/molecules30224355

**Published:** 2025-11-10

**Authors:** Shaobo Zhou, Linhong Yuan, Xiao Hu

**Affiliations:** 1 School of Science, Faculty of Engineering and Science, University of Greenwich, Medway Campus, Central Avenue, Chatham Maritime, Kent ME4 4TB, UK; 2 Beijing Key Laboratory of Environment and Aging, China-British Joint Laboratory of Nutrition Prevention and Control of Chronic Disease, School of Public Health, Capital Medical University, Beijing 100069, China; 3 Key Laboratory of Aquatic Product Processing, Ministry of Agriculture and Rural Affairs, South China Sea Fisheries Research Institute, Chinese Academy of Fishery Sciences, Guangzhou 510300, China; hnhuxiao@163.com

## 1. Introduction

Functional foods and dietary bioactives are moving from discovery to translation because we can now engineer structure and exposure together—the two levers that largely determine real-world efficacy. On the structure side, advances in drying, green/pressurised solvents, and enzyme-assisted fractionation allow precise tuning of chemotype (e.g., peptide-length distributions, glycosidic linkages, branching, particle morphology) so that physicochemical performance (rheology, texture, stability, release) is co-optimised with biological function (barrier interaction, receptor engagement, immunomodulation [1]. On the exposure side, harmonised in-vitro digestion/bioaccessibility models and omics readouts (targeted/untargeted metabolomics, glycomics, proteomics) tighten the link between what is consumed and what reaches mucosal, immune, and metabolic pathways—with growing attention to food matrix and meal timing effects that shape absorption, metabolism, and the microbiome [2,3]. Together, these advances enable batch-to-batch reproducibility, dose–response alignment to human pharmacokinetics, and claim-ready mechanisms that regulators and clinicians can evaluate—accelerating the path from laboratory characterisation to credible, scalable health applications across cardiometabolic, neurocognitive, and immune-inflammatory domains.

## 2. Coverage of Emerging Trends

This Special Issue, “Effects of Functional Foods and Dietary Bioactives on Human Health,” showcases the pipeline from isolation and characterisation through mechanistic understanding and, where feasible, application. The nine contributions span dairy and bee products, marine macroalgae, ginseng residues, and botanical polysaccharides or flavanones, using approaches ranging from rheology and textural analytics to cellular immunology and critical narrative reviews of disease contexts. Three recurring themes frame the collection: structure–function coupling, showing how extraction and processing alter molecular architecture and bioactivity; barrier biology and bioaccessibility, tracing the route from digestion to cellular uptake; and immunometabolic and microbiome-mediated mechanisms that connect foods to clinical endpoints.

The disciplinary breadth is both deliberate and complementary: food science and processing (tea, seaweed, ginseng), nutritional biochemistry and bioactives (peptides, polysaccharides, flavanones, 10-hydroxydecanoic acid), marine and algal bioproducts (proteins and polysaccharides), dairy and bee products (whey peptides and royal jelly lipids), and botanical medicine/pharmacology (e.g., Astragalus, oleanolic acid), with applications spanning oncology, neurology, and healthy ageing. Across this landscape, contributors addressed practical questions: how extraction or processing steps change structure and function; the bioaccessibility and digestibility of novel proteins and fibres; which mechanisms dominate (immunomodulation, antioxidant actions, microbiome shifts); whether food-derived molecules modulate clinically relevant pathways (e.g., IGF-1/PI3K/AKT/mTOR); and where the translation gaps remain in standardisation, dosing, safety and endpoints.

Illustrative highlights include whey-derived peptides modulating macrophage cytokines and pointing to immunoregulatory potential [Contribution 1]; a *Porphyra yezoensis* polysaccharide with defined rheology and antioxidant traits valuable for food design [Contribution 2]; oleanolic acid engaging the IGF-1→PI3K/AKT/mTOR axis in preclinical models and slowing cellular ageing [Contribution 3]; and drying temperature reshaping black tea flavour chemistry and bioactivity—demonstrating how unit operations steer health-linked phytochemicals [Contribution 4]. Protein work on brown *Durvillaea/Macrocystis* and green *Ulva* species, including a seaweed-derived mycoprotein, established measurable in vitro bioaccessibility/digestibility [Contribution 5], while extraction route determined the structure and functionality of ginseng-residue dietary fibre [Contribution 6]. Reviews consolidated anticancer immunometabolic actions of *Astragalus* polysaccharides [Contribution 7], clarified the therapeutic properties/targets of royal jelly 10-HDA [Contribution 8], and positioned flavanones as modulators of the gut microbiota with links to cognition along the gut–brain axis [Contribution 9].

## 3. Editorial Perspective and Future Outlook

Collectively, these papers reaffirm that process determines performance: extraction and drying conditions shape not only texture and palatability but also the integrity and bioactivity of molecules (e.g., *P. yezoensis* polysaccharide, ginseng fibre, black tea) [Contributions 2,4,6]. A barrier-aware mindset is now routine—simulated digestion and protein/fibre digestibility assays ground claims in likely human exposure (seaweed proteins, including a seaweed-derived mycoprotein) [Contribution 5]. Mechanistic reviews extend this to transporter pathways and mucosal–immune interfaces, clarifying how specific chemotypes act within complex systems (flavanones, 10-HDA, *Astragalus* polysaccharides) [Contributions 7–9]. A unifying thread is immunomodulation: dairy peptides can directly reprogramme macrophage signalling, while polysaccharides shape tumour microenvironments and hepatic immunity, highlighting translational routes from food components to clinically relevant endpoints [Contributions 1,7].

Within this frame, the Issue offers concrete exemplars of structure–exposure–mechanism alignment. Whey-derived peptides shifted THP-1 cytokine profiles toward an immunoregulatory phenotype—an innate immune mechanism for dairy bioactives [Contribution 1]. A *P. yezoensis* polysaccharide combined defined viscoelasticity with antioxidant capacity, illustrating how rheology-guided structuring can preserve function in product design [Contribution 2]. Oleanolic acid engaged IGF-1→PI3K/AKT/mTOR signalling to slow cellular ageing, pointing to nutrient–signal axes with geroscience potential [Contribution 3]. In tea processing, drying temperature remodelled sensory attributes, volatile chemistry and in vitro bioactivity—showing that unit operations can steer health-linked phytochemicals as well as flavour [Contribution 4]. Marine studies quantified bioaccessibility/digestibility of proteins from brown (*Durvillaea*, *Macrocystis*) and green (*Ulva*) seaweeds and a seaweed-derived mycoprotein, linking resource selection to exposure estimates [Contribution 5]. From an up-cycling perspective, extraction route (solvent/enzymatic) re-tuned the microstructure and techno-functionality of ginseng residue dietary fibre—process control as a lever for both nutrition and performance [Contribution 6]. Reviews integrated anticancer immunometabolic actions of *Astragalus* polysaccharides in liver cancer [Contribution 7], clarified targets and properties of royal jelly 10-HDA for future formulation/dosing [Contribution 8], and positioned flavanones as microbiota modulators with links to cognition along the gut–brain axis, nominating biomarkers for human trials [Contribution 9].

**Figure 1:**
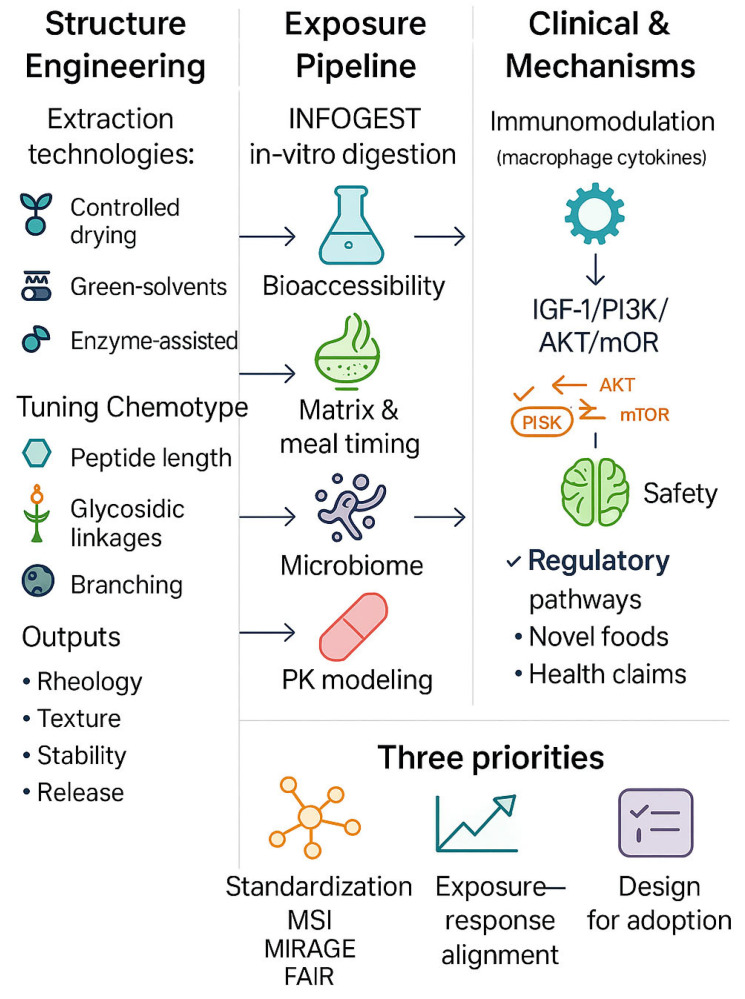
From discovery to translation: how structure and exposure drive the health effects of functional foods and dietary bioactives. Schematic overview illustrating the integrated path from processing-driven structural control to exposure modelling, mechanistic readouts, and translational endpoints. Advances in unit operations (e.g., drying, green solvents, enzyme-assisted fractionation) enable chemotype and matrix tuning to optimise physicochemical and biological performance. Harmonised in vitro digestion protocols (e.g., INFOGEST), food matrix and meal timing considerations, and bioaccessibility models strengthen exposure–response alignment. Mechanistic assays, including multi-omics, immunomodulation, and microbiome profiling, support clinically relevant evidence. The lower panel highlights three priorities to accelerate translation: (1) standardisation (MSI, MIRAGE, FAIR); (2) exposure–response alignment with pharmacokinetically informed dosing and validated endpoints; and (3) design-for-adoption including safety, shelf-life, and regulatory readiness. Left-to-right arrows denote the discovery-to-translation flow: Processing → Structure → Exposure → Mechanism → Evidence → Translation. Feedback loops indicate iteration (e.g., omics refining processing parameters, clinical readouts informing dose/formulation). Abbreviations: INFOGEST, internationally harmonised in vitro digestion protocol; MSI, Metabolomics Standards Initiative; MIRAGE, Minimum Information Required for A Glycomics Experiment; FAIR, Findable-Accessible-Interoperable-Reusable; PK, pharmacokinetics; GI, gastrointestinal.

To accelerate translation, three priorities are showed and emerged in this Special Issue (Figure 1). First, standardisation and analytics should deepen chemotype–phenotype mapping—through NMR or metabolomics and glycomics—to secure batch-to-batch equivalence and reproducible effects across sites and populations [4,5,6]. Second, exposure–response work should pair bioaccessibility/in vitro digestion models with pharmacokinetically informed human dosing, explicitly testing food matrix and meal timing effects and, wherever feasible, employing multi-omics endpoints aligned with validated clinical outcomes [1,7,8] [Contribution 7]. Third, design for use must optimise formats people will actually consume, consider price and shelf-life, quantify safety and interactions, and plan for regulatory pathways (e.g., novel foods; health claims), ideally with clinician–industry co-creation from the outset [2,3].

We thank all authors for their high-quality contributions, the reviewers for their rigorous and constructive feedback, and the editorial team for their support. The breadth of disciplines represented—food science, pharmacology, nutrition, and chemical biology—made this Special Issue a genuinely cross-sector effort.

## Data Availability

No new data were created or analyzed in this study. Data sharing is not applicable to this article.
